# Continuous EEG monitoring after brain tumor surgery

**DOI:** 10.1007/s00701-019-03982-6

**Published:** 2019-07-06

**Authors:** Kristin Elf, Elisabeth Ronne-Engström, Robert Semnic, Elham Rostami-Berglund, Jimmy Sundblom, Maria Zetterling

**Affiliations:** 1 Department of Neuroscience, Clinical Neurophysiology, Uppsala University, University Hospital, S-751 85 Uppsala, Sweden; 2 Department of Neuroscience, Neurosurgery, Uppsala University, University Hospital, S-751 85 Uppsala, Sweden; 3 Department of Surgical Sciences, Radiology, Uppsala University, S-751 85 Uppsala, Sweden

**Keywords:** Brain tumor surgery, Postoperative seizures, Non-convulsive seizures, EEG monitoring

## Abstract

**Background:**

Prolonged seizures generate cerebral hypoxia and increased intracranial pressure, resulting in an increased risk of neurological deterioration, increased long-term morbidity, and shorter survival. Seizures should be recognized early and treated promptly.

The aim of the study was to investigate the occurrence of postoperative seizures in patients undergoing craniotomy for primary brain tumors and to determine if non-convulsive seizures could explain some of the postoperative neurological deterioration that may occur after surgery.

**Methods:**

A single-center prospective study of 100 patients with suspected glioma. Participants were studied with EEG and video recording for at least 24 h after surgery.

**Results:**

Seven patients (7%) displayed seizure activity on EEG recording within 24 h after surgery and another two patients (2%) developed late seizures. One of the patients with early seizures also developed late seizures. In five patients (5%), there were non-convulsive seizures. Four of these patients had a combination of clinically overt and non-convulsive seizures and in one patient, all seizures were non-convulsive. The non-convulsive seizures accounted for the majority of total seizure time in those patients. Non-convulsive seizures could not explain six cases of unexpected postoperative neurological deterioration. Postoperative ischemic lesions were more common in patients with early postoperative seizures.

**Conclusions:**

Early seizures, including non-convulsive, occurred in 7% of our patients. Within this group, non-convulsive seizure activity had longer durations than clinically overt seizures, but only 1% of patients had exclusively non-convulsive seizures. Seizures were not associated with unexpected neurological deterioration.

**Electronic supplementary material:**

The online version of this article (10.1007/s00701-019-03982-6) contains supplementary material, which is available to authorized users.

## Introduction

Seizure activity is an energy-demanding process with a risk of secondary neuronal injury. Prolonged seizures generate cerebral hypoxia and increased intracranial pressure resulting in an increased risk of neurological deterioration, longer hospital stay, increased long-term morbidity, and shorter survival [10, 23, 26, 28]. It is thus crucial to find these patients at risk and treat them promptly.

Patients with brain tumors are prone to develop epileptic seizures [33]. Seizure is the first symptom in 80% of patients with low-grade gliomas (WHO grade II) [6], but is less common as the initial symptom in patients with high-grade gliomas (WHO grades III and IV) [37]. During the course of the disease, more than half of patients with high-grade gliomas develop seizures [37]. Seizures in patients with brain tumors are associated with progression of the disease and shorter survival [3, 6, 35].

Earlier studies have found varying rates of postoperative seizures after surgery for primary brain tumors, from 2% up to 34% [1, 10, 12, 16, 19, 20, 28, 31, 33, 37]. Postoperative seizures may occur whether or not the patients had seizures prior to surgery [10, 12, 20, 28], and the occurrence of postoperative seizures for the individual patient is difficult to predict. Today, the incidence of non-convulsive seizures after craniotomy for primary brain tumors is not known. Previous studies have shown that non-convulsive status epilepticus occurred in 2% of brain tumor patients [27]. In an intensive care setting, non-convulsive seizures were detected in 23% and non-convulsive status epilepticus in 12% of patients with brain tumors [8]. There has been no previous consecutive series of continuous EEG (cEEG) monitoring after brain tumor surgery and hence the true seizure occurrence of non-convulsive seizures is unknown.

After craniotomy for brain tumors, 7–17% [5, 7, 14, 25] of the patients suffer from a decline in the neurological function. In some of these patients, non-convulsive seizures may be a treatable cause of the deterioration.

The aim of the present study was to investigate the occurrence of postoperative seizures including non-convulsive seizures in patients undergoing craniotomy for primary brain tumors and to find out if non-convulsive seizures could explain some of the postoperative neurological deterioration that may occur after surgery.

## Methods and materials

### Patients

Adult patients (> 18 years of age) with a radiological suspicion of low- or high-grade glioma (WHO grades II–IV) planned for surgery at the Department of Neurosurgery, Uppsala University Hospital during the period 22 August 2016 to 7 December 2017 were prospectively included in the study. Informed consent was obtained prior to participation. The study was approved by the institutional ethics review board (2016/112).

Patients treated with anti-epileptic drugs (AED) prior to surgery continued with the ordinary dose. Intraoperative AED prophylaxis was administrated in awake surgery but in other cases according to the preferences of the surgeon. Before surgery, patients with epilepsy were optimized regarding seizure frequency with AED adjustment. No patient with an uncontrolled seizure situation was considered for surgery.

Patients went through preoperative magnetic resonance imaging (MRI), and postoperative MRI was performed within 48 h after surgery. In high-grade tumors, contrast enhancement on T1-weighted turbo spin echo sequences and, in low-grade tumors, high signal intensity on T2- fluid-attenuated inversion recovery (FLAIR) sequences were considered as tumor tissue. Postoperative MRI was used to identify any new postoperative ischemic areas.

In awake surgery, dexmedetomidine (Dexdor, Orion Pharma, Danderyd, Sweden) was administrated if required. Otherwise, general intravenous anesthesia with propofol (Diprivan, Ballerup, Denmark) and remifentanil (Remifentanil, TEVA Sweden AB, Helsingborg, Sweden) was used. 5-Aminolevulinic acid (5-ALA) (Gliolan, Medac Pharma, Varberg, Sweden) was used in patients with presumed high-grade (contrast enhancing) tumors if total resection was the goal of surgery.

All patients with postoperative seizures or neurological deterioration went through a CT scan to rule out a postoperative hematoma.

### EEG electrodes and postoperative continuous EEG video monitoring

After surgery, before dressing and with the patient still anesthetized, 11 needle electrodes (Technomed, Cephalon, Maastricht, the Netherlands) were attached to the scalp: bifrontal (F3, F4), bitemporal (T3, T4), biparietal (P3, P4), bioccipital (O1, O2), one midline (Cz), one ground, and one reference (in front of and behind Cz, respectively).

EEG and video monitoring (Nicolet Monitor, Natus Neuro, Middleton, USA) were initiated immediately upon arrival at postoperative care unit and continued until discharge to the general ward, as a rule, 24 h after surgery. EEG data were collected on the general hospital server utilizing the NicoletOne System, and interpretation was performed from a remote reading station with the NicoletOne Reader. All raw cEEG was read continuously, but EEG trends (amplitude integrated EEG and spectral edge) were used to facilitate interpretation and to reduce the risk of missing important episodes due to reader fatigue. The modified Salzburg Consensus Criteria were used for definition of non-convulsive seizures and status epilepticus [24]. The overall terminology and classification applied was the American Clinical Neurophysiology Society’s critical care standardized terminology [18].

### Statistics

To calculate differences between groups, Mann-Whitney *U* test for continuous and categorical variables and Fisher exact two-tailed test for proportions were used in Statistica, version 13.2 (StatSoft, Inc. Tulsa, OK, USA). A *p* value < 0.05 was considered statistically significant.

## Results

### Clinical data

One hundred patients, 59 men and 41 women, underwent tumor resection. The mean age ± SD was 53.5 (± 16.2) years. Eleven patients went through awake surgery and in 25 patients, perioperative neurophysiology monitoring was carried out. In 21 patients, 5-ALA was used. The most common preoperative symptoms were epileptic seizures (52%) followed by cognitive deficits (25%). In 89% of the cases, the tumors involved the frontal and/ or temporal lobes. The histopathological diagnoses were high-grade gliomas WHO grades III–IV in 69% and WHO grade II gliomas in 24% of the patients. Preoperative symptoms and tumor locations are presented in Table 1 and the final diagnosis in Table 2. Median (25th–75th percentile) preoperative tumor volume was 32.4 cm^3^ (11.2–74.5). Median (25th–75th percentile) resection grade was 96.5% (72.5–100).

**Table 1:** Preoperative symptoms and tumor locations in 100 patients

**Symptom**	**%**
Seizures	52
Cognitive deficit	25
Language disturbances	14
Motor deficit	12
Visual field deficit	7
Gait-coordination disturbance	4
Headache	19
Asymptomatic	5
**Patients with**	***N***
No symptom	5
One symptom	63
Two symptoms	26
Three symptoms	6
**Tumor location**	**%**
Frontal	33
Temporal	27
Parietal	4
Occipital	2
Insular*	12
Frontal + corpus callosum/gyrus cinguli	8
Frontal-parietal-temporal	1
Temporal-occipital	4
Parietal-temporal	4
Parietal-occipital	4
Midline	1

*Fronto-insular *n* = 2, temporal-insular *n* = 1, fronto-temporal-insular *n* = 8, fronto-temporal-insular + central *n* = 1

**Table 2:** Tumor diagnosis in 100 patients

Tumor diagnosis	*N*	%
WHO grade IV		50
Glioblastoma	48	
Gliosarcoma	2	
WHO grade III		19
Anaplastic astrocytoma	11	
Anaplastic oligodendroglioma	6	
Anaplastic Ependymoma	1	
Anaplastic pleomorphic xantoastroctoma	1	
WHO grade II		24
Astrocytoma	9	
Oligodendroglioma	13	
Ependymoma	1	
Not classified	1	
WHO grade I		1
Pilocytic astrocytoma	1	
Metastasis		2
Adenocarcinoma	1	
Gastric carcinoma	1	4
Other		4
B cell lymphoma	1	
Unclassified	3	

### Preoperative epilepsy and AED

Fifty-two percent of patients had seizures preoperatively. In another 3% of patients, there was suspicion, but not a definitive diagnosis of epileptic seizures. Fifty-three percent used AED before surgery. In one patient, the indication for prescription of AED was a pain. Epileptic manifestations and AED treatments are presented in Online Resource. Three patients were diagnosed with epilepsy in childhood. Two of these were still on anti-epileptic drugs and showed a sporadic and low frequency of focal seizures with impaired consciousness. In one patient, epilepsy terminated in childhood and no AED had been used since.

### Intraoperative AED prophylaxis and intraoperative seizures

Sixteen percent of patients received intraoperative AED prophylaxis with fosphenytoin or levetiracetam. One of these developed EEG-verified seizures during the first 24 h after surgery.

Two patients experienced intraoperative seizures. One patient developed bilateral tonic-clonic seizures after subcortical motor stimulation during general anesthesia and the other patient showed focal seizures during awake surgery.

### Seizures within 24 h after surgery

Seven percent of patients displayed seizure activity on cEEG recording within 24 h after surgery. Tables 3 and 4 summarize the findings in these patients. Two of the 7 patients had a preoperative diagnosis of epilepsy and one of the 7 patients was treated with AED.

**Table 3:** Patients with ictal activity on EEG recording within 24 h of surgery and with late seizures > 24 h after surgery

Pat nr M/F Age (year)	Preop seizure	Preop AED	Tumor location	Diagnosis	5-ALA	Tumor volume (cm^3^)	Postop ischemic lesion	Grade of resection (%)	Seizure starts in relation to surgery	Semiology seizures postop	EEG ictal start	EEG ictal activity			EEG monitoring time
												No	Duration total (min:sec)	Duration Non-convulsive Ictal activity (min:sec)	
1 M 63	No	No	Temp-Ins L	GBM	No	70.2	No	62	14 h 36 min	Focal to bilat tonic-clonic	Front L	1.	2:4		19 h 23 min
2 F 45	No	No	Front-Par-Occ L	B cell lymphoma	Yes^	7.3	No	100	Between 6 h 8 min and 6 h 35 min°	1. Subclinical to bilat tonic-clonic 2. Subclinical to focal motor	Par L	1.	7:38 to 33:56°	6:19 to 32:37	69 h 28 min
												2.	25:21	4:1	
3 M 66	Yes Ch	No	Front R	GBM	No	73.1	No	97	3 h 42 min	1. Subclinical to bilat tonic-clonic	Front R	1.	1:28	0:20	43 h 50 min
										2. Subclinical to bilat tonic-clonic		2.	4:3	2:50	
4 M 75	No	No	Temp R	GBM	No	53.2	Yes	100	1 h 39 min	1. Subclinical	Front R	1.	12:55	12:55	7 days 17 h 47 min
										2. Subclinical to bilat tonic-clonic		2.	6:1	4:51	
										3. Focal Mot chin		3.	1:25		
5 F 51	Yes	Yes	Front-Temp-Ins L	Oligo II	No	41.1	Yes	92	5 h 56 min	1. Subclinical	Temp L	1.	0:50	0:50	23 h 9 min + 80 h 3 min
										2. Subclinical to Focal Mot clonic R hand and arm		2.	1:52	1:10	
												3.	1:11	0:24	
6 M 65	No	No	CC Midline	Unclass	No	16	No	73		Focal with impaired awareness	Bilat		†	†	88 h 4 min
7 M 71	No	No	Occ R	Metastasis Adenoca	No	15.3	Yes	100	10 min	Subclinical	Occ R	8.	42:25	42:25	22 h 41 min
Late seizures															
5	See patient 5 above.								For information about late seizures, see online resource.						
8 M 68	Yes	Yes	Front L	Oligo III	No	11.6	No	100	120 h	Focal motor	L		7:28	No video	22 h 39 min + 9 days 12 h
9 M 70	Yes Ch + Ad	Yes	Temp R	GBM	Yes	43.9	No	98	96 h	Bilat tonic-clonic	Front R Sharp Waves		Rec started after seizures had finished.		15 h 37 min + 18 h 15 min

*Pat*, patient; *Nr*, number; *M*, male; *F*, female; *Preop*, preoperative; *AED*, anti-epileptic drugs; *5-ALA*, 5-aminolevulinic acid; *Postop*, postoperatively; *No*, number; *Min*, minutes; *Sec*, seconds; *h*, hour; *Subclin*, subclinical; *Temp*, temporal; *Ins*, insular; *L*, left; *GBM*, Glioblastoma Multiforme WHO grade IV; *Bilat*, bilateral; Front, frontal; *Par*, parietal; *Occ*, occipital; ^5-ALA was used because the radiological suspicion was high-grade glioma; °the recording was stopped due to an examination and the ictal activity started during this time; *Ch*, childhood; *R*, right; *Mot*, motor; *Oligo II/III*, oligodendroglioma WHO grade II/III; *CC*, corpus callosum; *Unclass*, unclassified; †EEG showed bilateral periodic discharges (PDs) for long periods. The patient was sedated and the clinical semiology faded concomitantly with PDs disappearing from EEG. Bilateral PDs appeared again but as the patient was quietly at rest; no clinical testing was performed. Therefore, the seizure duration cannot be measured or estimated; *Adenoca*, adenocarcinoma; *Ad*, adult; *Rec*, recording

**Table 4:** A summary of the patients with ictal activity on continuous EEG or clinically suspected seizures

Patient	Postoperative seizures	Ictal activity in EEG	Semiology	Complicated clinical course*	Postop ischemic lesion	Preoperative EP	AED
1	Early < 24	Yes	Overt	Yes	No	No	No
2	Early < 24	Yes	Non-convulsive and overt	Yes	No	No	No
3	Early < 24	Yes	Non-convulsive and overt	No	No	Yes Childhood	No
4	Early < 24	Yes	Non-convulsive and overt	Yes	Yes	No	No
5	Early and late > 24 h	Yes	Non-convulsive and overt	Yes	Yes	Yes	Yes
6	Early	Yes	Non-convulsive and subtle	Yes	No	No	No
7	Early < 24 h	Yes	Non-convulsive	No	Yes	No	No
8	Late > 24 h	Yes	Overt	Yes	No	Yes	Yes
9	Late > 24 h	No	Overt	Yes	No	Yes Childhood + adult	Yes
10	Clin suspected <24 h	No	Focal motor	No	No	Yes	Yes
11	Clin suspected <24 h	No	Focal motor	No	No	Yes	Yes
12	Clin suspected <24 h	No	Focal motor	No	No	Yes	Yes
13	Clin suspected <24 h	No	Impaired consciousness	No	No	Yes	Yes

*A complicated clinical course was defined as prolonged stay in the intensive or intermediate ward or readmission to these because of complications

*Postop*, postoperatively; *Clin*, clinically

### Non-convulsive seizures

Seizure activity in the EEG without any clinical signs of seizures (non-convulsive seizure) was detected in five (5%) of patients. For details, see Table 3. Mostly, non-convulsive seizures preceded the onset of clinical seizures but there were isolated non-convulsive seizures as well. In one patient, there was 42 min of unnoticed non-convulsive seizures. In one patient, the recording was stopped due to an examination and the seizure activity started during this time. Therefore, the seizure time is presented as an interval, the shortest to the longest time possible. The summarized duration of non-convulsive seizure represented 72–78% of total seizure time.

### Late postoperative seizures in patients who were seizure free during the initial 24 h after surgery

Two patients were seizure free during the initial 24-h EEG monitoring period but developed late seizures (see Tables 3 and 4). In addition, one of the patients with early seizures also developed late seizures that are described in Online Resource.

### Clinical suspicion of postoperative seizures but no seizure activity on EEG

In four patients, there were clinical suspicions of seizures during the EEG monitoring time but no concomitant ictal EEG pattern was found. The symptoms in those patients were suspected of focal motor seizures in three patients and focal seizures with impaired consciousness in one patient (Table 4).

### Clinical factors

There were no differences in tumor volumes, resection grades, tumor locations, diagnosis, or the use of 5-ALA for patients who developed seizures postoperatively and for those who did not. On postoperative MRI, a larger proportion of patients with early postoperative seizures showed ischemic lesions (3/7, 43%), compared to patients without early seizures (11/93, 12%). An epilepsy diagnosis earlier in life, was present in 2 of 7 (29%) patients with early postoperative seizures and in 1 of the 93 (1.1%) patients without early postoperative seizures.

### Postoperative neurological deterioration

Six patients deteriorated postoperatively during the EEG monitoring time. The symptoms in these patients were paresis in one hand (*n* = 1), dysphasia (*n* = 2), dysphasia, hemiparesis and decreased the level of consciousness (*n* = 1), dilatation of one pupil and hemiparesis (*n* = 1), and confusion (*n* = 1). CT scanning did not reveal any explanation and non-convulsive seizures were considered a possible cause of deterioration at that time. However, none of these patients exhibited seizure activity on EEG recording, and a specific period of postictal slowing could not be identified. Therefore, the postoperative neurological deterioration in those cases could neither be explained by non-convulsive seizures nor by subtle, non-convulsive seizures followed by postictal symptoms.

### Postoperative ischemia, hematoma, and second surgery

In 14 patients (14%), postoperative MRI showed a new ischemic lesion. Nine of these patients (64%) deteriorated neurologically after surgery but there was complete regression of postoperative neurological deficits in 8 patients and almost complete regression in 1 patient. Three patients underwent a second surgery. One patient developed a postoperative hematoma in the surgical field which was evacuated 24 h after the primary surgery when a hemiparesis developed. Another patient was reintubated directly after the primary surgery due to a decreased level of consciousness and received an intraparenchymal pressure monitoring device. The third patient displayed a generalized tonic-clonic seizure and decreased the level of consciousness. A CT scanning revealed a distant hematoma in the posterior fossa and the patient was subjected to an external intraventricular drainage procedure.

## Discussion

In this study, we monitored the occurrence of seizure activity on continous EEG recording the first 24 h after surgery for primary brain tumors in 100 consecutive cases. The aim was to estimate the occurrence of convulsive and non-convulsive seizures and to determine if they were related to postoperative neurological deterioration. We found that seizures occurred in 7% of patients within 24 h after surgery. Another 2% of patients developed late seizures after 24 h which required prolonged EEG monitoring time and one of the patients with early seizures also developed late seizures.

After surgery for brain tumors, it is not uncommon that the surgeon encounters a patient who has deteriorated neurologically. As a standard, an immediate CT scan is performed to rule out an expansive hematoma requiring evacuation. In some cases, such as surgery in connection to eloquent areas, the deterioration of a specific neurological function is expected. However, in other cases, the reason for the deterioration is unclear, and non-convulsive seizures are considered a possible cause. Since an acute EEG usually is difficult to arrange outside office time, the question of whether the patient should be treated with AED with potentially serious side effects [2], without prior EEG may appear. This study provides information about the occurrence of non-convulsive seizures in a consecutive group of patients with assumed primary brain tumors, information that could be useful in the above-described situation.

This is to our knowledge the first prospective study in which EEG monitoring was used after surgery for brain tumors in consecutive material. We found that altogether 9% of patients had seizures postoperatively. This is lower than what usually is reported in the literature, probably because we studied a consecutive material. Earlier studies reporting seizure incidence after brain tumor surgery have either been performed on high-risk groups in the intensive care setting [8, 13, 22, 29] or are retrospective studies without systematically EEG monitoring [1, 3, 6, 10, 16, 33–35]. The incidence of convulsive seizures after craniotomy for primary brain tumors varies in different studies from 2 to 44%, [1, 10, 12, 13, 16, 19, 20, 28, 31, 33, 37]. This variation could be explained by differences in patient selection, different observation times postoperatively, or differences in postoperative routines and thereby the ability to detect seizures.

This is also the first study to report of non-convulsive seizures in the acute phase after surgery of brain tumors. Previous studies were done in epilepsy-prone patients and/or patients in the intensive care unit [8, 13, 22, 29]. These studies showed that non-convulsive status epilepticus occurred in 2% of brain tumor patients [27]. In the intensive care unit, non-convulsive seizures were detected in 23% and non-convulsive status epilepticus in 12% of patients with brain tumors [8]. In the neurointensive care unit, 18–34% of patients suffered from non-convulsive status epilepticus [22, 29], and non-convulsive seizures were an important cause of an unexplained decrease in the level of consciousness [8]. We found a lower frequency of non-convulsive seizures (5%). This seizure incidence is likely more useful in estimating the occurrence and the risk of postoperative seizes in brain tumor patients in general because our patient series was consecutively collected without any selection bias.

One question in our study was if seizure activity could explain some of the postoperative worsening of neurological function. However, among the six patients with an early unexplained postoperative decline in neurological function, no seizure activity was present in the cEEG recordings. This finding supports the conclusions that, in case of unexpected neurological deterioration, other causes than non-convulsive seizures should be excluded and treatment with anti-epileptic drugs may not be justified without prior EEG diagnosis. Another four patients displayed episodes of clinically suspect seizures but without seizure activity in the EEG. In such cases, shivering, tremor, and drug-induced myoclonia may be misinterpreted as a seizure. There is a possibility is that we underdiagnosed non-convulsive seizures because the nine surface electrodes used were too sparse to record very focal seizure activity. It is recommended to use at least 16 electrodes [17], but for this postoperative study of brain tumor patients, we gave priority to single-use needle electrodes in order to ensure sterility and to secure electrode contact under the head bandage. Therefore, we chose to reduce the number of electrodes to 11.

Studies on patients during neurointensive care have shown a discrepancy in the occurrence of seizure activity recorded by intracerebral electrodes (ICE) and surface electrodes with a higher incidence of electrographic seizures detected by ICE compared to scalp EEG [9, 36]. We did not use ICE, but such recordings may have been useful since two of our patients with unexplained deterioration showed patterns related to seizure activity registered by ICE.

Before surgery, low-grade glioma [19], cortical location [3, 6], temporal lobe involvement [3], and oligodendroglioma subtypes [6] have been identified as risk factors for preoperative seizures. For the individual patient, the occurrence of epileptic seizures acutely after craniotomy for a brain tumor is difficult to predict. Higher age [33, 34], small tumors [33], complete resections [33], and parietal lobe involvement [3] or diencephalic location [33] have been related to postoperative seizures. Regarding the influence of tumor grade, there are diverging results [10, 33]. The occurrence of seizures before surgery [1, 13, 19, 28, 34], especially if there is a long history of seizures [30] or if the seizures are focal [30] or uncontrolled [3], was positively correlated with seizures postoperatively. However, other studies showed no relationship between preoperative seizures or treatment with anti-epileptic drugs and postoperative seizures [4, 10, 20, 33].

Except for a tendency for more postoperative ischemic lesions in patients with early postoperative seizures, we did not discern differences in clinical factors between patients with or without seizures after surgery. This may be because the number of patients with postoperative seizures was too small to reliably calculate risk factors for this group.

A recent meta-analysis showed a beneficial effect for AED prophylaxis on reducing the incidence of early postoperative seizures [21] but today, there is no general consensus for AED prophylaxis in patients with a brain tumor and no prior seizures [11, 15, 32, 38]. The three patients in this study who went through a second monitoring due to recurrent late seizures were on treatment with AED before surgery, but only one of the seven patients with early seizures used AED preoperatively.

To conclude, early postoperative seizures, including non-convulsive seizures, occurred in 7% of our patients. Within this group, non-convulsive seizures had longer durations than clinically overt seizures. However, non-convulsive seizures were not the cause of unexplained postoperative deterioration in this patient series. In cases of unclear deterioration, other treatable causes should be excluded.

## Electronic Supplementary Material

ESM 1 (DOCX 21 kb)

## Abbreviations

AED: Anti-epileptic drug
5-ALA: 5-Aminolevulinic acid
CT: Computed tomography
cEEG: Continuous electro-encephalography
ICE: Intracerebral EEG
ICP: Intracranial pressure
MRI: Magnetic resonance imaging
WHO: World Health Organization
