# Supplementary material for: Continuous EEG monitoring after brain tumor surgery
Source: Acta Neurochir (Wien). 2019 Jul 6;161(9):1835–43. doi: 10.1007/s00701-019-03982-6 (PMC6704081; doi:10.1007/s00701-019-03982-6)
Supplement: Supplementary file 1 — (DOCX 21 kb) [file 701_2019_3982_MOESM1_ESM.docx]

**Online Resource 1,** **Tables 1-3**

Early continuous EEG monitoring after brain tumour surgery, Acta Neurochirurgica

Kristin Elf^1^, Elisabeth Ronne-Engström^2^, Robert Semnic ^3^, Elham Rostami-Berglund ^2^, Jimmy Sundblom ^2^, Maria Zetterling ^2^

1. Department of Neuroscience, Clinical Neurophysiology, 2. Department of Neuroscience, Neurosurgery, 3. Department of Surgical Sciences, Radiology.

Uppsala University, University Hospital, S-751 85 Uppsala, Sweden.

Corresponding Author: [maria.zetterling@neuro.uu.se](mailto:maria.zetterling@neuro.uu.se)

| **Number of epileptic manifestations** | **Type of epileptic manifestations** | **N** | **%** |
| --- | --- | --- | --- |
| One | Generalized | 11 | 21 |
|  | Focal with impaired consciousness | 10 | 19 |
|  | Focal motor | 7 | 13 |
|  | Focal speech | 2 | 4 |
|  |  |  |  |
| Two | Focal with impaired consciousness + secondary generalized | 8 | 15 |
|  | Focal motor + secondary generalized | 3 | 6 |
|  | Focal speech + secondary generalized | 3 | 6 |
|  |  |  |  |
|  | Focal with impaired consciousness + focal motor | 1 | 2 |
|  | Focal speech + visual field | 2 | 4 |
|  | Focal speech + motor | 1 | 2 |
|  |  |  |  |
| Three | Focal speech + focal motor + secondary generalized | 1 | 2 |
|  | Focal speech + focal sensory + secondary generalized | 1 | 2 |
|  | Focal with impaired consciousness + focal speech + focal motor | 2 | 4 |
| **Number of seizures** | | |  |
| One occasion | | 15 | 29 |
| Repeated seizures | | 37 | 71 |

**Table 1:** The epileptic manifestations and number of seizures in the 52 patients with pre-operative epilepsy.

| **Number of antiepileptic drugs** | **N** | **Drug(s) (n)** |
| --- | --- | --- |
| Monotherapy | 45 | Levotirazetam (37) |
|  |  | Lamotrigine (7) |
|  |  | Carbamazepine (1) |
| Two drugs | 5 | Levotirazetam + Phenytoin (2) |
|  |  | Lamotrigine + Levotirazetam (1) |
|  |  | Gabapentin + Phenytoin (1) |
|  |  | Lamotrigine + Carbamazepine (1) |
| Three drugs | 2 | Clonazepam + Lamotrigin + Phenytoin + (1) |
|  |  | Gabapentin, Levotirazetam, Carbamazepine (1) |
| Four drugs | 1 | Valproate, Lacosamide, Levetiracetam, Perampanel (1) |

**Table 2**: Antiepileptic drugs used in the patients preoperatively.

| Seizure number | Semiology | Seizure start on EEG | Total duration (min: sec) | Subclinical* duration  (min: sec) |
| --- | --- | --- | --- | --- |
| 4 | Focal motor, clonic movements starting in chin, corner of the mouth spreading to head via R versive and eye-deviation to R, then clonic movements of R arm and hand | Temp L | 1:33 | - |
| 5 | As number 4 | Temp L | 1:31 | - |
| 6 | As number 4 | Temp L and Front L | 2:42 | - |
| 7-9 | Clonic movements of R chin and cheek | Temp Left | 0:20 | - |
| 10 | Focal motor (R side clonic movements) to subclin* to focal motor | Temp L | 3:52 | 1:22 |
| 11 | Focal mot (R side clonic movements) to subclin* to focal motor | Temp L | 3:49 | 1:12 |
| 12 | Subclin* start to focal motor (R side clonic movements) then switching between these two another three times | Temp L | 8:31 | 2:36  (19s + 1:06 + 44s +27s) |
| 13 | Focal motor (R side clonic movements) | Temp L | 2:09 | - |
| 14 | Subclin to focal motor (R side clonic movements) to subclin | Temp L | 6:11 | 2:31 |

**Table 3.** Seizures in patient 5 during the second monitoring period.

Min = minutes, Sec = seconds, R = right, L = left, Temp = temporal, Front = frontal, Subclin = subclinical.

*Subclinical in this context may be temporal semiology with silence; sometimes the patient was pulling or moving the bed linen, this can either be purposeless, compatible with temporal semiology, but the patient may be trying to get rid of the duvet, feeling warm after the clonic seizure. No other subtle temporal semiology such as oral automatisms or dystonia could be seen in the video.
